# Sex-related differences in motor learning and performance

**DOI:** 10.1186/1744-9081-6-74

**Published:** 2010-12-23

**Authors:** Pablo Moreno-Briseño, Rosalinda Díaz, Aurelio Campos-Romo, Juan Fernandez-Ruiz

**Affiliations:** 1 Departamento de Fisiología, Facultad de Medicina, Universidad Nacional Autónoma de México. México; 2 Facultad de Psicología, Universidad Veracruzana, Xalapa, Veracruz, México

## Abstract

Gender differences have been shown across many domains, and motor skills are no exception. One of the most robust findings is a significant sex difference in throwing accuracy, which reflects the advantage of men in targeting abilities. However, little is known about the basis of this difference. To try to dissect possible mechanisms involved in this difference, here we tested for gender variations in a prism adaptation throwing task. We tested 154 subjects in a visuomotor prism adaptation task that discriminates between motor performance, visuomotor adaptation and negative aftereffects. Our results corroborate men's significant better throwing accuracy, although there were no adaptation differences between genders. In contrast, women showed significant larger negative aftereffects, which could be explained by a larger contribution of spatial alignment. These results suggest that different learning mechanisms, like strategic calibration and spatial alignment, may have different contributions in men and women.

## Background

Among the most robust examples of differences between men and women is the better throwing accuracy shown by men [1,2]. Together with a better spatial ability, it has been suggested that this gender difference arise since early human ages, when men went out hunting, while women stayed with the children while gathering food or making manual labor[3].

Whatever its origins, gender differences for throwing accuracy can be found even in children, suggesting that the gender effect is independent of age [4]. In studies involving adults, the throwing accuracy male advantage has been shown to be independent of different paper-and-pencil spatial tasks [5], or mental rotation, a task in which male outperform women [6,7]. Although practice was initially considered as a possible gender difference factor in throwing accuracy [4], later analyses suggested that the difference stood even after the effects of sports history were considered [5].

So, it is possible that men and women have different visuomotor approaches on how they make throws, and that such difference results in different gender accuracies. To explore this possibility we decided to test men and women in a prism adaptation task that involves throwing balls at a target [8]. This task has the added benefit that it makes possible to separate visuomotor performance from visuomotor learning [9-11]. For example, patient populations like Parkinson's disease (PD) or Huntington's disease (HD) show significant impairments in visuomotor performance as measured by the large variance showed in their baseline throws; however, their visuomotor adaptation rate remains largely intact during the prism adaptation phase of the task [12].

## Materials and methods

### Participants

In this study, one hundred and fifty four healthy volunteers participated. All subjects were right handed. There were 76 men (mean age 39.2 ± 13.5 SDM; range from 18 to 65 y/o), and 78 female (mean age 39.7 ± 13.1 SDM; range from 18 to 65 y/o). A two-tailed Student's *t *test showed that there were no age differences between groups (t = 0.2254, df = 150, p = 0.8220). The experimental procedures followed were in accordance with the ethical standards of the committees on human experimentation of the Universidad Nacional Autónoma de México. In addition, all subjects gave their informed consent prior to the experiments in accordance with the Helsinki Declaration.

### Procedure

The general prism adaptation procedure used in this experiment has been described elsewhere [8,13], and it follows the throwing technique developed earlier [10]. Subjects viewed the target binocularly through 30 diopter Fresnel 3 M Press-on plastic lenses (3 M Health Care, Specialties Division, St. Paul, MN, USA). During the task subjects threw clay balls (weight: 10 g) at the target, which was a 10-cm × 10-cm cross drawn on a large sheet of parcel paper centered at shoulder level 2 m in front of them. The position at which the balls made an impact on or around the target was marked immediately after each throw with a marker pen by an experimenter standing outside the visual field of the subject. Subjects stood without changing their foot position during performance of the task, their head was unrestrained, and no directions were given about trunk, shoulder, or head-neck posture. However, they were instructed to make only overhand throws.

The experiment followed three phases previously described [8,10]. During the first phase, named PRE, a baseline error of the throwing performance was obtained by having subjects throw 25 balls to the target before they donned prisms. After donning 30 diopters prisms, during phase PRI, subjects were instructed to throw 25 more balls with the same arm and in the same way. After removing the prisms, during phase POS, subjects threw 25 more balls again with the same arm and in the same way. Subjects had an unobstructed view of the target during the entire session, but were instructed not to look down at their hands as they collected the balls from a tray next to them during throws. The location of each impact was plotted sequentially by trial number. Impacts to the left of the target were plotted as negative values and impacts to the right as positive values. Two additional values were calculated from the collected data. First, an adaptation measure was obtained from phase PRI by subtracting the distance to the center of the ball's impact on the final throw from that on the initial throw. Second, an aftereffect measure was defined as the distance from the center of the first throw after removing the prisms (phase POS). It is important to note that the first throw in the POST phase is a real measurement of aftereffect not contaminated by any kind of expectation or feedback, including possible corollary discharge information.

The statistical analyses included an F-test to evaluate the equality of variances assumption. Since in all instances this criterion was not met, a t-Test for unequal samples followed the F-test. To test for differences in the adaptation rate we used the General Linear Model for Repeated Measures as described in the results section.

## Results

**Figure 1:**
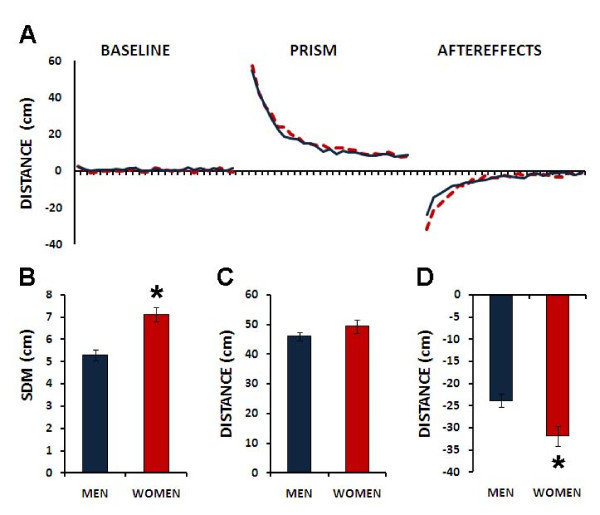
**Prism adaptation performance by gender**. A. Error (impact distance to target in cm) during baseline, prism, and after prism withdrawing for men (solid lines) and women (dashed lines). B. Motor performance measured by the standard deviation of the mean (SDM) on both groups. C. Adaptation (in cm). D. Aftereffects (in cm). * = p < 0.05.

Results from the three phases can be seen in Figure 1A. There are no differences in the error distance means between both groups in the baseline. However, the standard deviation analysis showed that men were significantly more accurate than women (Figure 1B). An F-Test for the significance of the difference between the variances of the two Samples was significant (F = 1.71; p = 0.01). A two-tailed t-Test for unequal sample variances showed significant accuracy differences between men and women (df = 143.9; t = -4.5: p < 0.001). To further analyze the baseline performance of both groups, we also computed the total absolute errors, and the variable errors. The absolute baseline errors mean for women was 5.72 ± 0.13 SEM, while for men was 4.37 ± 0.1 SEM. A two-tailed Student t-test for independent variables show that there were significant gender differences (p < 0.001). A similar analysis for the Variable errors also point out significant differences (women X = 7 ± 0.31 SEM, men X = 5.6 ± 0.24 SEM; p < 0.001).

The analysis of the adaptation measure showed no differences between the two groups (Figure 1C). An F-Test for the significance of the difference between the variances of the two samples was significant (F = 1.45; p = 0.05). A two tailed t-Test for unequal sample variances showed that there were no significant accuracy differences between men and women (df = 148.3; t = -1.4: p = 0.14).

To test if there were differences in the adaptation rate, the adaptation phase of both groups was analyzed using a General Linear Model Repeated Measures analysis. Using gender and throw number as independent variables the multivariate test (Pillai's trace) showed that there were throw effects (F = 105.4, p < 0.001), but there was not an interaction between throws and gender (F = 1, p = 0.467). The between-subjects analysis showed that there were no gender differences (F = 0.729, p = 0.395).

Finally, an analysis of the gender difference in the aftereffect showed significant differences between men and women (Figure 1D). An F-Test for the significance of the difference between the variances of the two Samples was significant (F = 1.46; p = 0.05). A two tailed t-Test for unequal sample variances showed significant accuracy differences between men and women (df = 148.1; t = 3.6: p < 0.001).

## Discussion

The better throwing accuracy shown by males was an expected result, since it has been corroborated several times in different laboratories. The results obtained during the prism adaptation phase show that there were no gender differences on the error reduction of this visuomotor learning task. However, there was a significant difference in the aftereffects shown by males and females. Contrary to the better performance shown by males in the baseline, women showed larger aftereffects once the prisms were removed.

There is only one previous article on gender differences during a prism adaptation throwing task [14]. The authors tested 25 men and 30 women on a 10-diopter prism adaptation throwing task. However, since they were only interested in how variable were the throws, they only analyzed absolute values. Their analysis showed, as expected, that men were more accurate in all three phases. A report on heavy and light alcohol drinkers that included an analysis on gender differences during prism adaptation suggested that males show larger adaptation to prisms [15], however, a similar study did not find any gender effect [16]. In those studies, however, subjects were not tested with a throwing paradigm, so it is difficult to know if the throwing gender differences would apply to them.

It has been proposed that there are two processes at work during prism adaptation: strategic calibration and spatial alignment [17]. For other names that have been proposed for similar concepts see [18]. Strategic calibration helps to adjust motor commands in a given space that does not necessarily translate to other effectors, or conditions, and does not affect the spatial relationship between the motor and visual modalities [17]. Strategic calibration does not lead to aftereffects. In contrast, spatial alignment does produce a rearrangement in the motor and visual systems relationship, probably to adjust for long term changes like body growth. Spatial alignment leads to large aftereffects [17].

Trying to implement a cognitive control to the adaptation process appear to engage more the strategic calibration process, although spatial alignment seems to continue in parallel [19]. For instance, it has been shown that when subjects are more aware of prisms, there are smaller aftereffects than when they are not aware [20]. Therefore, it could be possible that during adaptation a larger strategic calibration contribution in males resulted in a smaller aftereffect. However, the same adaptation rate shown by both populations suggest that this is not the case, since a larger contribution of strategic calibration results in faster adaptation rates [21]. Another option would be that women show a larger expression of the spatial alignment once the prisms are withdrawn. As mentioned above, larger aftereffects are shown when subjects do not try to implement strategic calibrations [20], or when cognitive mechanisms are compromised, like during normal aging or Alzheimer dementia [22,23]. Therefore, it could be possible that the sex-related aftereffect difference that we found would be the result of a larger participation of spatial alignment in women that results in larger aftereffects.

## Limitations

The results suggest a dissociation between the better throwing performance shown by men, and the larger aftereffects produced by women once the prisms were withdrawn. However the experiment was not specifically designed to test for differences between strategic calibration and spatial alignment. Therefore, it would be important to implement such an experiment to dissect the contributions of those processes in each gender.

## Conclusions

Men and women show a different pattern of results in a prism adaptation task. The results obtained from each population suggest a different contribution of strategic calibration and spatial alignment, which are two processes involved in motor control and motor learning.

## Competing interests

The authors declare that they have no competing interests.

## Authors' contributions

Authors PMB and JFR designed and directed the entire project and performed the statistical analyses. PMB, RD and ACR tested the experimental subjects. All authors contributed to, and approved the final manuscript.
